# Addition of synthetic polymer in the freezing solution of mesenchymal stem cells from equine adipose tissue as a future perspective for reducing of DMSO concentration

**DOI:** 10.29374/2527-2179.bjvm002523

**Published:** 2023-12-27

**Authors:** Cátia Nascimento, Márcia Viviane Alves Saraiva, Vitoria Mattos Pereira, Danielle Cristina Calado de Brito, Francisco Léo Nascimento de Aguiar, Benner Geraldo Alves, Kelly Cristine Santos Roballo, José Ricardo de Figueiredo, Carlos Eduardo Ambrósio, Ana Paula Ribeiro Rodrigues

**Affiliations:** 1 Veterinarian, MSc. Laboratório de Manipulação de Oócitos e Folículos Pré-Antrais Ovarianos (LAMOFOPA), Faculdade de Medicina Veterinária, Universidade Estadual do Ceará, Fortaleza, CE, Brazil;; 2 Veterinarian, DSc. Universidade Federal Rural do Semiárido (UFERSA), Mossoró, RN, Brazil;; 3 Veterinarian, MSc. Departamento de Medicina Veterinária, Faculdade de Zootecnia e Engenharia de Alimentos, Universidade de São Paulo, SP, Brazil;; 4 Biologist, DSc. LAMOFOPA, Faculdade de Medicina Veterinária, Universidade Estadual do Ceará, Fortaleza, CE, Brazil;; 5 Veterinarian, DSc. Instituto Federal de Educação, Ciência e Tecnologia da Paraíba, Sousa, PB, Brazil;; 6 Veterinarian, DSc. Laboratório de Biologia da Reprodução, Universidade Federal de Uberlândia, Uberlândia, MG, Brazil;; 7 Veterinarian, DSc. Departamento de Medicina Veterinária, Faculdade de Zootecnia e Engenharia de Alimentos, Universidade de São Paulo, SP, Brazil;; 8 Veterinarian, DSc. LAMOFOPA, Faculdade de Medicina Veterinária, Universidade Estadual do Ceará, Fortaleza, CE, Brazil;; 9 Veterinarian, DSc. Departamento de Medicina Veterinária, Faculdade de Zootecnia e Engenharia de Alimentos, Universidade de São Paulo, SP, Brazil;; 10 Veterinarian, DSc. LAMOFOPA, Faculdade de Medicina Veterinária, Universidade Estadual do Ceará, Fortaleza, CE, Brazil.

**Keywords:** regenerative therapy, horse, trehalose, superCool X-1000, cryopreservation, terapia regenerativa, cavalo, trealose, superCool X-1000, criopreservação

## Abstract

The regenerative therapies with stem cells (SC) has been increased by the cryopreservation, permitting cell storage for extended periods. However, the permeating cryoprotectant agents (CPAs) such as dimethylsulfoxide (DMSO) can cause severe adverse effects. Therefore, this study evaluated equine mesenchymal stem cells derived from adipose tissue (eAT-MSCs) in fresh (Control) or after slow freezing (SF) in different freezing solutions (FS). The FS comprise DMSO and non-permeating CPAs [Trehalose (T) and the SuperCool X-1000 (X)] in association or not, totalizing seven different FS: (DMSO; T; X; DMSO+T; DMSO+X; T+X, and DMSO+T+X). Before and after cryopreservation were evaluated, viability, colony forming unit (CFU), and cellular differentiation capacity. After freezing-thawing, the viability of the eAT-MSCs reduced (P< 0.05) in all treatments compared to the control. However, the viability of frozen eAT-MSCs in DMSO (80.3 ± 0.6) was superior (P<0.05) to the other FS. Regarding CFU, no difference (P>0.05) was observed between fresh and frozen cells. After freezing-thawing, the eAT-MSCs showed osteogenic, chondrogenic, and adipogenic lineages differentiation potential. Nonetheless, despite the significative reduction in the osteogenic differentiation capacity between fresh and frozen cells, no differences (P > 0.05) were observed among FS. Furthermore, the number of chondrogenic differentiation cells frozen in DMSO+X solution reduced (P<0.05) comparing to the control, without differ (P>0.05) to the other FS. The adipogenic differentiation did not differ (P>0.05) among treatments. In conclusion, although these findings confirm the success of DMSO to cryopreserve eAT-MSCs, the Super Cool X-1000 could be a promise to reduce the DMSO concentration in a FS.

## Resumo

As terapias regenerativas com células-tronco (CT) têm sido incrementadas pela criopreservação, permitindo o armazenamento celular. No entanto, os agentes crioprotetores (ACPs) penetrantes, como DMSO, podem causar efeitos adversos graves. Portanto, este estudo avaliou células-tronco mesenquimais equinas derivadas de tecido adiposo (CTM-TAe) *in natura* (Controle) ou após congelamento lento (CL) em diferentes soluções de congelamento (SC). As SCs compreendem DMSO e ACPs não permeáveis [Trealose (T) e o SuperCool X-1000 (X)] associados ou não: (DMSO; T; X; DMSO+T; DMSO+X; T +X e DMSO+T+X). Antes e após a criopreservação foram avaliados, viabilidade, unidade formadora de colônia (UFC) e capacidade de diferenciação celular. Após o congelamento-descongelamento, a viabilidade das CTM-TAe reduziu (P< 0,05) em todos os tratamentos em relação ao controle. Entretanto, a viabilidade das CTM-TAe congeladas em DMSO (80,3 ± 0,6) foi superior (P<0,05) às demais SC. Em relação às UFC, não houve diferença (P>0,05) entre células frescas e congeladas. Após congelamento-descongelamento, as CTM-TAe apresentaram potencial de diferenciação de linhagens osteogênicas, condrogênicas e adipogênicas. No entanto, apesar da redução significativa na capacidade de diferenciação osteogênica entre células frescas e congeladas, não foram observadas diferenças (P > 0,05) entre SCs. Além disso, o número de células de diferenciação condrogênica congeladas em solução de DMSO+X reduziu (P<0,05) em relação ao controle, sem diferir (P>0,05) das demais SCs. A diferenciação adipogênica não diferiu (P>0,05) entre os tratamentos. Em conclusão, embora esses achados confirmem o sucesso do DMSO para criopreservar CTM-TAe, o Super Cool X-1000 pode ser uma promessa para reduzir a concentração de DMSO.

## Introduction

The skeletal muscle diseases have produced a significant loss for the equine industry, emerging as the main orthopedic problems for this species. For example, a high rate of the caregiver in the affected animals (Trela et al., 2014). This problem is mainly caused due to the low capacity of regeneration of the cartilage and tendinous tissue in horses (Khodadadi et al., 2012). In this scenario, among the promising therapeutic tools used for the treatment of the muscle-skeletal diseases, is notably the use of stem cells (SC), mainly the mesenchymal (MSCs), which consists of multipotent cells that can be obtained from several tissues of adult animals and develop an essential role in the tissue regeneration (Vidane et al., 2013), and immunomodulation. Recently, previous studies demonstrated in vivo the beneficial utilization of MSCs in several diseases, such as cervical osteoarthritis in humans (Jo et al., 2014), chronic kidney disease in cats (Vidane et al., 2017), spinal cord lesions in dogs (Feitosa et al., 2017) and the tendinous lesion in equines (Durgam & Stewart, 2017).

Give the importance and necessity of the utilization of animal models (Gonçalves et al., 2014), the equine is considered as an attractive option for the research of the orthopedic affections, once the horse demonstrates proportional similarities in size, weight to carry, and types of articulations with the human being (Ranera et al., 2011). Additionally, the cellular therapy in equines (Hackett & Fortier, 2011) has been great application’s potential in the veterinarian clinical practice, due to the fast development of studies towards MSCs (Sharma et al., 2014). Hence, the American food and drug agency concluded that the horse is the most appropriated animal model to test the clinical effects of MSCs, mainly considering articular lesions (Mitchell et al., 2015; Ranera et al., 2011).

In this context, the equine adipose tissue-derived mesenchymal stem cells (eAT-MSCs) have been the core source of cell acquisition in horses, owing to several factors, such as: availability, minimal invasive harvesting procedure, massive quantity of obtained cells, besides the capacity and efficiency of these cells to differentiate in several lineages (Frisbie & Smith, 2010). However, so that MSCs have a disseminated utilization is critical to the creation of cryopreserved cell banking, which will be cryostored in a vast amount, available to the therapeutical application. The implantation of SC banking is feasible when considered some critical aspects such as viability, proliferative capacity, and cellular differentiation capacity, which should be maintained after the procedures of freezing/thawing. Therefore, these factors have been the focus of scientific interest of several investigators (Ginani et al., 2012; Mambelli et al., 2009; Renzi et al., 2012), when it comes to cryopreservation protocols of stem cells.

For the cryopreservation of the biological material, is imperative the utilization of cryoprotectant agents (CPAs), which can be classified into two types: penetrating and non-penetrating. This CPAs help to minimize the cryoinjuries induced during the different phases of the cryopreservation process (Castro et al., 2011). Among the penetrating CPAs available, the DMSO is the most used mainly for the cryopreservation of stem cells (Sousa, 2014). However, depending on the concentration and temperature, as the physiological, the DMSO offers severe risks to the cells due to its toxicity (Elliott et al., 2017). Previous studies have shown that 10% of DMSO is the most used concentration for the freezing of MSCs (Chabot et al., 2017; Gurruchaga et al., 2015). Hence, to avoid cell damages and other complications after thawing, it is necessary the removal of the DMSO, through many washes, followed by centrifugation of SC, which leads to a considerable reduction in the number of available cells for the application (Mitchell et al., 2015). In this regard, with the goal to minimize the toxicity, as well as the loss of MSCs, the strategies of association of DMSO with non-penetrating CPAs, or even the substitution of penetrating by non-penetrating CPAs have been investigated (Shu et al., 2014).

Among the non-penetrating CPAs, the disaccharides, for instance, the trehalose has been used with success for the freezing of MSCs of the umbilical cord (Motta et al., 2014), and human peripheral blood (Chen et al., 2016; Martinetti et al., 2017). The trehalose is a non-toxic molecule able to avoid the osmotic shock, protecting the cellular membrane of protein denaturation and destabilization induced during freezing (Solocinski et al., 2017). Additionally, another promising alternative is the synthetic polymers, for instance the alcohol polivinilic copolymer or SuperCool X-1000, produced towards analogous substances of antifreeze glycoprotein’s (Deller et al., 2014; Wowk et al., 2000), which protect fishes of the low-temperature regions (-2 °C) or extremophilic microorganisms (Caruso et al., 2018). These polymers decreased the freezing point, and support in the reduction of the ice crystals formation (Deller et al., 2014). Therefore, aiming to reduce the toxicity caused by DMSO, as well as the loss of MSCs during the penetration CPAs remove process, this study has the goal to evaluate the efficiency of in vitro freeze eAT-MSCs in different freezing solutions comprised by the association or not of penetrating (DMSO), and non-penetrating CPAs (Trehalose and SuperCool X-1000).

## Materials and methods

The present study was approved and performed under the guidance of the Animal use ethics committee of the Faculty of Animal Science and Food Engineering, University of São Paulo - FZEA/USP (#6083170317).

### Reagents and antibodies

All the reagents and media used during the experiments were acquired from Gibco (Gibco®, Invitrogen, USA) unless otherwise stated. The pH of the medium was equilibrated for 7.4, and the osmolality was adjusted for 280 mOsm/kg. For the immunophenotyping, it was used the following primary antibodies: mouse monoclonal CD90 (SC – 6071, Santa Cruz, Dallas, USA), mouse monoclonal CD44 (MCA1082GA, AbDSerotek, Raleigh, NC, USA), mouse MHC (Major Histocompatibility Complex) class I monomorphic (MCA1086GA, AbDSerotek, Raleigh, NC, USA), mouse monoclonal CD86 (NB100-77815, BD Pharmigen, São Paulo, Brazil) e mouse MHC Class II Monomorphic (MCA1085GA, AbDSerotek, Raleigh, NC, USA). For the secondary antibodies, it was used the Alexa Fluor® 488 rabbit anti-goat IgG (11078, Serotek, Raleigh, NC, USA) and the polyclonal goat anti-mouse immunoglobulins/FITC (F-0479, Dako, Bath, UK).

### Animals

Four healthy mixed-breeding mares with age varying between 5 and 13 years were submitted to harvesting of the adipose tissue for further isolation of the eAT-MSCs. The animals were previously evaluated for the presence of physiological alterations, and submitted to hematologic evaluation for the confirmation of healthiness, and for the exclusion of the compulsory notification pathologies following World Organization for Animal Health – OIE, (i.e., equine infectious anemia virus or glanders).

### Adipose tissue harvest

The protocol of harvesting of adipose tissue was performed as described (Vidal et al., 2007). Briefly, the animals were firstly sedated with detomidine hydrochloride (2.2 mg/kg of the body weight), administrated intravenously, and then, an area of 10 cm^2^ in the base of the tail, under the dorsal gluteus muscle was trichotomized and prepared aseptically by an application of a local anesthetic (2% lidocaine without vasoconstrictor), in which a fragment of ± 2 grams of adipose tissue were harvested. Therefore, the samples of adipose tissue were washed in a phosphate buffer saline (PBS) solution added with 1% of penicillin, 1% of streptomycin, and 1% of amphotericin B (wash solution). After that, the sample was transported to the laboratory at 4 °C, and then, submitted to an additional four washes using the same solution.

### Isolation and in vitro culture of mesenchymal stem cells

After wash, the samples of adipose tissue were fragmented with stainless steel, transferred to tubes containing a solution of PBS added with 75 UI/ml of collagenase type I (Gibco®, Invitrogen, USA), 1% of penicillin, 1% streptomycin, 1% amphotericin B for enzymatic digestion, and were incubated at 38.3 °C, in an atmosphere of 5% CO2 for 1.5 hours, and homogenized at each 10 min. Soon after, the samples of each animal were centrifuged at 1700 rpm for 5 min, and the supernatant was discharged, and the pellet was resuspended and cultured in vitro in 3 ml of culture medium composed by minimum essential medium alpha medium (α-MEM) (Gibco®, Invitrogen, USA), supplemented with 10% of fetal bovine serum (SFB, Gibco®, Invitrogen, USA), 1% of glutamine (Gibco®, Invitrogen, USA), 1% of non-essential amino acids (Gibco®, Invitrogen, USA), 1% of penicillin, 1% streptomycin (Gibco®, Invitrogen, USA), and 1% of amphotericin B (Gibco®, Invitrogen, USA) namely α-MEM+. The resulting cellular samples were plated in cell culture flasks (25 mm), and incubated in a 38.3 °C for 14 days, corresponding to the primary cell culture, or P0.

During the primary cell culture, the partial replacement of the medium (1 ml) was performed at every 2-3 days in the first week and a full replacement of the medium (3 ml) from the second week. In each medium replacement, the cells were checked by its morphology, capacity of adherence in culture flasks, and capacity of multiplication. Since the confluence of 90% was obtained, the cells were trypsinized and re-plated (a procedure called first passage or P1). For the trypsinization, the medium α-MEM+ was removed, and 3 ml of TrypleTM Express Enzyme (ThermoFisher Scientific, USA) was added to the flasks, incubated for 5 min, and then it was evaluated to an optical microscope to confirm the cell unattachment. Then, 2 ml of α-MEM+ were added into the flask for inactivation of trypsin and follow-up cell plating. The process of cell expansion was performed until the second passage or P2.

### Immunophenotypic characterization

To confirm and characterize immunophenotypically the eAT-MSCs, some aliquots of 5x10^5^ cells in P2, fresh or non-frozen were suspended in 200 μl of PBS, added with 0.1% of bovine serum albumin (BSA), and submitted to an unspecific block solution (PBS and 1% of BSA) for 1 h in a room temperature. After blockage, the cells were incubated for 1 hour in a dark chamber at room temperature for evaluation of superficial markers, which were positive for mesenchymal stem cells` goat anti-CD90 (1:100), and mouse anti-CD44 (1:100), and the negative superficial markers for hematopoietic stem cells [primary antibodies, mouse anti-CD86 (1:100), mouse anti-MHC class I (1:100), and mouse anti-MHC class II (1:100)]. Additionally, at the same temperature mentioned above, the incubation of secondary antibodies was performed with each of the primary antibodies [anti-goat Alexia 488 (1:300), and anti-mouse Dako FITC (1:300)] for 1 hour. Finally, the cells were fixed in paraformaldehyde at 2% for 10 min and then submitted to a cytometry flow analysis (FACS ARIA, BD). As a negative control, aliquots of both superficial cell markers (positive and negative) were submitted only to a solution with PBS and secondary antibodies (Thitilertdecha et al., 2020).

### Experimental design

The eAT-MSCs at P2 of each animal was divided into eight aliquots of 1x10^6^ cells/ml. Then, an aliquot of fresh or non-frozen cells was used as a control group and immediately submitted to endpoint evaluations such as viability, colony forming unit (CFU), and cellular differentiation. The other aliquots (n = 7) were destined to slow freezing with different freezing solutions (FS), comprised by α-MEM+ added of penetrating (DMSO) or non-penetrating CPAs (Trehalose and SuperCool X-1000), with or without association, as represented at Table 1.

**Table 1:** Different freezing solutions to cryopreserve eAT-MSCs.

**Freezing solutions composition**	**Simplified nomenclature**
α-MEM^+^ + 10% DMSO	DMSO
α-MEM^+^ + 60 mM Trehalose	T
α-MEM^+^ + 1% SuperCool X–1000	X
α-MEM^+^ + 10% DMSO + 60 mM Trehalose	DMSO+T
α-MEM^+^ + 10% DMSO + 1% SuperCool X–1000	DMSO+X
α-MEM^+^ + 60 mM Trehalose + 1% SuperCool X–1000	T+X
α-MEM^+^ + 10% DMSO + 60 mM Trehalose + 1% SuperCool X–1000	DMSO+T+X

T = Trehalose; X = X-1000.

### Slow freezing, storage, and thawing of mesenchymal stem cells

The slow freezing protocol used was adapted from Mambelli et al. (2009). The aliquots of eAT-MSCs were collected in cryotubes of 2 ml and exposed to different FS in room temperature for 20 min. After the exposure, the cryotubes containing eAT-MSCs were put in a Mrs. Frosty (Ref. 5100-0001 - Nalgene®, Brazil), and maintained in an ultra-freezer for 24 hours, which permitted a gradual reduction of the temperature of 1 °C/min until reach -80 °C. After that, the cryotubes were transferred to a liquid nitrogen tank (LN_2_) and stored for five days.

For thawing, the cryotubes of each FS were removed from LN_2_, maintained at room temperature for 1 min, and then, plunged in a water bath for 3 min at 37 °C. For CPAs removal, the cells were transferred for a falcon tube of 15 ml, containing 1 ml of α-MEM+, and then, centrifuged at 1600 rpm for 5 min. The supernatant was discharged, and 1 ml of α-MEM+ was added to a pellet and homogenized. Thus, the aliquots were evaluated immediately for cellular viability. Only the cells originated from freezing treatments having viability rates of 40% were submitted to evaluation of CFU and cellular differentiation.

### Cell viability

The cellular viability of the eAT-MSCs was evaluated using the vital dye Trypan blue (Sigma-Aldrich, MO, USA). Therefore, ten μl of the dye was added to a fraction of 10 μl of the suspension containing fresh cells or from each freezing treatment. Afterward, ten μl of the cell suspension were led to a Neubauer chamber for counting the number of living cells. This methodology was performed as previously described (Renzi et al., 2012), in which the cells were considered viable or non-viable hen labeled or not by the Trypan blue dye, respectively.

### Colony forming unit (CFU)

To evaluate the capacity of the colony formation of the eAT-MSCs before and after freezing, aliquots of 2 ml containing 1x10^4^ cells/ml in P2 were cultured in triplicate in 6 well plates for 13 days, with a full refresh of the medium performed at every three days. After the culture, the medium was removed, and the cells were washed twice in PBS for 5 min for each wash and fixed in 4% paraformaldehyde during 30 min. After, the cells were submitted to three consecutive washes in PBS aiming to remove the fixative and stained with 0.1% Giemsa for 15 min at room temperature. After staining, the counting of the number of colonies with more than 50 cells was performed (Vidal et al., 2007).

### Differentiation analysis

Fresh (control) and frozen eAT-MSCs that demonstrated cell viability superior to 40% were submitted to in vitro culture for evaluation of the differentiation potential of the lineages adipogenic, osteogenic, and chondrogenic, using commercial differentiation kits (Gibco®-Life Technologies, USA), which were specific for each lineage, in accordance with the manufacture protocol. For higher security, it was used a negative control that was composed of cell culture only with the presence of α-MEM+.

For evaluation of the differentiation of osteogenic and adipogenic cells, 1 x 10^5^ cells/ml eAT-MSCs were cultured in 12 well plates containing 1 ml of α-MEM+ until reach the confluence of 60%, for then, the addition of the specific media differentiation. In the case of adipogenic differentiation, it was used the StemPro® Adipogenesis Differentiation Kit (Gibco-Life Technologies, USA), added of 1% of penicillin, 1% streptomycin, in which the cells remained in culture for more 14 days. After this period, the cells were washed in PBS, fixed in paraformaldehyde 4%, stained with Oil Red O for 5 min, and evaluated for optical microscopy for the presence of intracytoplasmic lipidic vesicles. The determination of the potential adipose intensity of differentiation was performed by a scoring system in the Oil Red O staining, as described in Table 2 (Burk et al., 2013; Fülber et al., 2016).

**Table 2:** Semi-quantitative scoring system used in the evaluation eAT-MSCs after adipogenic differentiation.

**Values (%) and references aspects**		
**Score**	**% of differentiated cells**	**Size and arrangement of lipids droplets**
0	0-5	No droplets
1	> 5-50	Isolated and small
2	> 50-80	Medium sized
3	> 80-100	Predominantly large
**Values (%) and references found**		
**Treatments**	**Average score**	**Size and arrangement of lipids droplets**
Control	2	Medium sized
DMSO	2	Medium sized
DMSO+X	1.5	Isolated and small
DMSO+T+X	2.5	Medium sized

T = Trehalose; X = X-1000.

For the osteogenic differentiation, the cells were cultured in StemPro® Osteogenesis Differentiation Kit (Gibco-Life Technologies, USA), with 1% of penicillin, 1% streptomycin, for 21 days. After this period, the cells were washed and fixed, as described previously, and stained with Alizarin Red for 15 min for the evaluation of the formation of calcium matrix. To quantification, the differentiation area and stained by the Alizarin Red labeling was calculated using the Image J program (Loci, University of Wisconsin, Madison), as previously described (Fülber et al., 2016).

In contrast with the earlier analysis, the chondrogenic differentiation was performed in a cell pellet, which was obtained after centrifugation of 1 x 10^5^ cells/ml eAT-MSCs, and cultured in tubes of 15 ml with 0.5 ml of α-MEM+ for four days. From that moment, the addition of the medium StemPro Chondrogenesis Differentiation Kit (Gibco-Life Technologies, USA), with 1% of penicillin, 1% streptomycin, and then, the cells were cultured for more 21 days. After the culture period, the medium was discharged, and the performed chondrogenic pellet was fixed for 30 min in paraformaldehyde 4%, dehydrated in ethanol, clarified in xylene, and included in paraffin. Serial sections (5 μm) of the pellet were cut and stained with Alcian Blue for identification of proteoglycans. The quantification of the intensity of the chondrogenic differentiation was performed by the determination of an index between the total area of the image and the differentiated area (µm2), as previously described (Burk et al., 2013), and demonstrated by the following formula:


Index = differentiated area/ total area of the image (1)

### Statistical analysis

The data were analyzed by the software Sigma Plot 11.0 (Systat Software Inc, San Jose, California, USA). Firstly, it was applied the Shapiro-Wilk to determine the normal distribution of the data. The osteogenic and chondrogenic differentiation that did not have a normal distribution were ranked and submitted to ANOVA or Student’ t-test. The viability and the number of CFU were compared among the treatments by ANOVA followed by posthoc Student-Newman-Keuls test. The data were demonstrated as the mean ± standard error of the mean, and the results were considered significant when P < 0.05.

## Results

### Morphological analyses of eAT-MSCs

**Figure 1:**
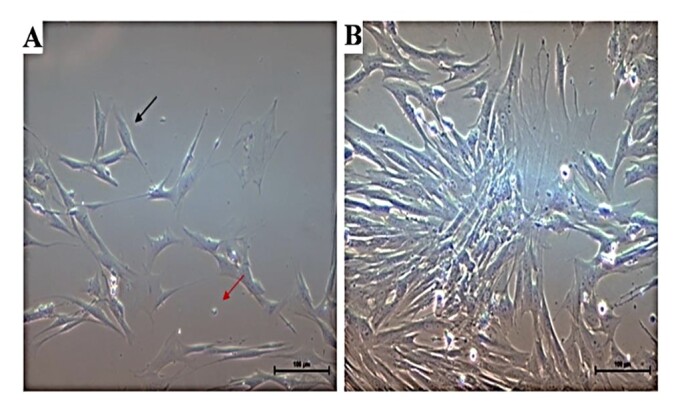
The representation of the primary culture of eAT-MSCs. (A) Adherent cells in the first 48 hours of culture; (B) The 80-90% of confluence after 14 days of culture. The black arrow indicates an adherent cell with a fusiform format and the red arrow indicates a non-adherent cell with a round shaped form. Scale bar = 100 μm.

The primary cell culture obtained after processing of adipose tissue was comprised by a heterogeneous cellular population, which demonstrated adherent cells to the plastic surface of culture flasks, with a fusiform aspect, whereas the non-adherent cells presented a round shaped format (Figure 1). After 48 hours of plating, it was observed a predominance of adherent cells with fusiform or fibroblastoide morphology; characteristic described as a pattern for mesenchymal stem cells.

### Immunophenotypic assay

**Figure 2:**
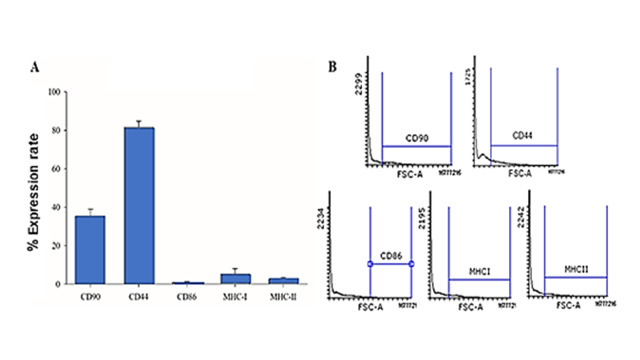
Immunophenotypic analysis of fresh eAT-MSCs, showed the reactivity, (A, B) with high positive expression of mesenchymal stem cells markers (CD90 and CD44) but almost no expression for hematopoietic markes (CD86, MHC-1 and MHC-II).

The profile analysis of the superficial markers by flow cytometry of the fresh cells in P2 demonstrated the expression of the consistent immunophenotypic characteristic of MSCs (Figure 2). The results indicated that the cells expressed high levels of cellular markers, i.e., CD90 and CD44, which are present in MSCs. In contrast, the cells did not express the common cell markers of SCs from hematopoietic sources, such as the CD86, MHC I, and MHC II.

### Viability of eAT-MSCs post - cryopreservation

**Figure 3:**
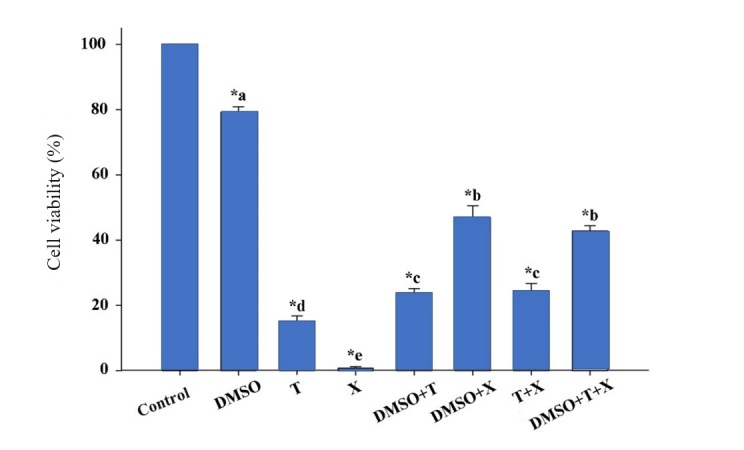
Mean (± SEM) percentages of cell viability of eAT-MSCs before and after freezing. *Differs from Control. Different lowercase letters indicate significant difference among FS (P <0.05). T = Trehalose and X = SuperCool X-1000.

The viability of frozen eAT-MSCs in all the treatments demonstrated a significant reduction when compared to fresh cells (Figure 3). However, the percentage of viable frozen eAT-MSCs in the presence only of DMSO was significantly superior (80.3 ± 0.6) to the observed in the other treatments. Frozen cells only in the presence of X-1000 (0.7 ± 0.4) or Trehalose (15.0 ± 1.7) demonstrated the lowest rates of viability post freezing (P < 0.05). Nevertheless, when the DMSO was associated with X-1000 (DMSO + X), the rate of cell viability was superior to 40% (47.0 ± 3.5), demonstrating then a beneficial effect of X-1000 when associated with DMSO. Moreover, the cell viability was similar (P > 0.05) between the combinations of DMSO + X-1000 and DMSO + Trehalose + X-1000 (42.8 ± 1.5), In contrast, it was verified that the association of DMSO + Trehalose (23.9 ± 1.2) or X-1000 + Trehalose (24.5 ± 2.2) did not favor the post-freezing cellular viability.

### CFU assay

**Figure 4:**
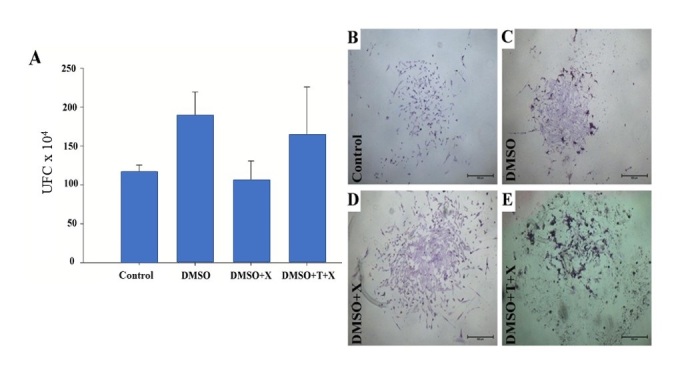
Colony-forming unit of the eAT-MSCs. Mean (± SEM) percentages of CFU from the control and post-freezing groups of treatments with viability above 40% (A). Representative images of colony formation in the control group (B), and in different freezing solutions (C, D, and E). Staining: Giemsa; Scale bar = 100 μm. T = Trehalose; X = SuperCool X-1000.

After freezing, the eAT-MSCs from the freeing solutions that demonstrated cellular viability superior of 40% (DMSO, DMSO+X-1000 and DMSO+Trehalose+X-1000) were capable of multiple and form colonies similarly to the observed in the control group and did not differ (P > 0.05) among the groups (Figure 4).

### Differentiation analysis post-cryopreservation

**Figure 5:**
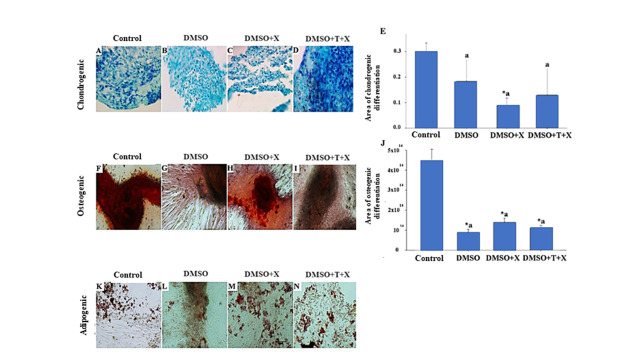
Representative figure of the differentiation potential of eAT-MSCs after culture of post-freezing groups of treatments with viability above 40%. Differentiation chondrogenic (A, B, C, and D), with the presence of proteoglycans. Differentiation osteogenic (F, G, H, and I), with the formation of a rich bone matrix. Differentiation adipogenic (K, L, M, and N), with the presence of fat droplets. Mean (± SEM) percentages of differentiation chondrogenic (E) and osteogenic (J). *Differs from Control. Different lowercase letters indicate significant difference among FS (P <0.05). T = Trehalose; X = SuperCool X-1000.

Fresh and frozen eAT-MSCs had morphological alterations by the chondrogenic, osteogenic, and adipogenic differentiation. The chondrogenic differentiation was confirmed by the presence of proteoglycans, stained by Alcian Blue in the histological sections of the cellular pellets (Figure 5A-D). The data demonstrate that in the presence of only DMSO or the association of DMSO + Trehalose + X-1000, the eAT-MSCs had a chondrogenic differentiation potential similar (P > 0.05) to the control. On the other hand, the differentiation potential of the frozen cells in DMSO + X-1000 was inferior (P < 0.05) to control. However, no significant difference was observed between the different freezing treatments (P > 0.05).

Concerning the osteogenic differentiation, the confirmation was detected by the deposition of Calcium phosphate in the extracellular matrix stained by the Alizarin Red (Figure 5F-I). It was observed a reduction (P < 0.05) in the osteogenic potential intensity in the frozen eAT-MSCs in all treatments when compared to the control. However, among the different tested FS, no differences (P > 0.05) were observed (Figure 5J).

The alteration of cell morphology confirmed the adipogenic lineage from fusiform to polygonal and by the presence of intracytoplasmic lipidic vesicles stained by Oil Red O (Figure 5K-N). The frozen cells in all the treatments did not differ in the differentiation capability of the adipocytes about the control group lineages (Table 2).

## Discussion

We investigate the effect of the cryopreserved eAT-MSCs in the presence of different freezing solutions, which were comprised by penetrating and non-penetrating CPAs, with or without association, on the viability, maintenance of the colony formation capacity, and the in vitro differentiation potential.

Initially, the isolated cells from the equine adipose tissue grew in a single layer, and assumed a fibroblastoid morphology, following the first criteria required to categorize the MSCs culture (Bourin et al., 2013; Dominici et al., 2006). Thereafter, during the culture, the cells demonstrated high capacity of adhesion in a plastic, an excellent capacity of multiplication, and at the end of the passages (P2), these cells expressed cell superficial markers, which matched with the classification of MSCs (positives: CD90 and CD44, and negatives for hematopoietic stem cells: CD86, MHC I and MHC II). Additionally, the cells showed an outstanding differentiation potential on the three induced lineages: osteogenic, adipogenic, and chondrogenic (Zomer et al., 2015).

After freezing, all the treatments significantly reduced the viability of the eAT-MSCs when compared with the control, probably due to the osmotic stress, to the toxicity of the CPAs, or to the injuries related with the internal procedure of cryopreservation (Meryman, 2007; Ribeiro et al., 2012). Although the DMSO has been known by its toxicity, more than 80% of the freeze eAT-MSCs in an FS containing only the cryoprotector remained viable after thawing. These findings are under previous reports in equines, which showed that the utilization of DMSO in the FS of AT-MSCs had an elevation in the cell viability rate (84 ± 3.9%) post-thawing (Renzi et al., 2012). The DMSO has a more widely used penetrating cryoprotectant of MSCs from the different tissues, such as equine umbilical cord (Maia et al., 2017), human bone medulla (Kaplan et al., 2017), and adipose tissue of sheep and mouse (Renzi et al., 2012). This cryoprotectant shows a low molecular weight (78.13 g/mol), that is why it can quickly pass through the plasmatic membrane, blinds to water molecules in solution, and then, blocks the hydric efflux of the cytoplasm during the freezing process. Consequently, the DMSO reduces the massive cellular dehydration, and maintains the stability of the saline concentrations and the pH, decreasing the formation as well as the size of the ice crystals in the extracellular and intracellular media (Sauer-Heilborn et al., 2004; Windrum et al., 2005).

On the other hand of the use of DMSO, the utilization of isolated Trehalose or X-100 was not capable of promoting a satisfactory cryoprotective effect of eAT-MSCs during the freezing, once both CPAs reduced the viability post-thawing (15.0 ± 1.7% and 0.7 ± 0.4%) drastically, respectively. This result could be because when used alone, the non-penetrating CPAs were not capable of avoiding the intracellular ice crystal formation (Elliott et al., 2017). However, the association of DMSO+X-1000 (47.0 ± 3.5%) or DMSO +Trehalose+X-1000 (42.8 ± 1.5%), guaranteed the viability of the half of cells, confirming the importance of the combination between penetrating and non-penetrating CPAs (Motta et al., 2014; Rodrigues et al., 2008; Solocinski et al., 2017). The action of both types of CPAs was complimentary; while the DMSO controls the reduction of the formation of intracellular ice crystals, the non-penetrating CPAs help in the maintenance of the stability of cellular membrane and reduces the osmotic shock (De Rosa et al., 2009; Renzi et al., 2012).

Differently of the penetrating CPAs that interfere in the cryopreservation by interacting with water, particularly the X-1000, acts in the nucleation point of ice in growth, inhibiting the blinding of other molecules of water in the flat surface, and avoiding then the formation of spicules in the ice crystals, which could cause great cell damages during the process. In this perspective, studies showed that the polymer X-1000 associated to the other CPAs, such as ethylene glycol and sucrose, or even the DMSO, resulting in a high viability after the vitrification of mouse embryos (72%: Badrzadeh et al., 2010), and preantral follicles of monkeys (80%: Ting et al., 2013), respectively. Additionally, a recent study demonstrated that the vitrification of enclosed preantral follicles on caprine ovarian tissue in the presence of X-1000 polymer maintained the follicular morphology after seven days of in vitro culture, similarly to observed for new or non-vitrified follicles (Montano Vizcarra et al., 2020).

By the other side, despite the association of penetrating and non-penetrating CPAs has been recommended, in the present study the association of DMSO+Trehalose resulted in a low percentage of viable cells (23.9% ± 1.2), which can be attributed to an inadequate of the concentrations of DMSO (10%), in association of trehalose (60 mM). Recent findings demonstrated elevated rates of cellular viability post-thawing when the DMSO was associated with the trehalose in concentrations of 100 mM and 300 mM (85% and 75%), respectively in human hepatic carcinoma cells (Solocinski et al., 2017), and 500 mM (75%) in eAT-MSCs (Renzi et al., 2012). Based on these findings, we believed that other combinations of the non-penetrating CPAs (X-1000 and Trehalose) in concentrations more elevated than the tested in the present study, which will permit a reduction in the concentration of DMSO, aiming to avoid the potentially deleterious effects in the use of high concentrations of penetrating CPAs.

Considering the low cellular viability obtained post freezing in the presence of trehalose (15.0 ± 1.7%), X-1000 (0.7 ± 0.4%), DMSO+Trehalose (23.9 ± 1.2), or Trehalose+X-1000 (24.5 ± 2.2), only the freezing cells in the FS, which resulted in a viability equal or superior to 40% were destined for the evaluation of the colony formation capacity and differentiation. The results demonstrated that the FS containing DMSO, DMSO+X-1000, or DMSO+Trehalose+X-1000, did not show differences in the potential to form colonies with the control. These findings agreed to a study performed with MSCs in an umbilical cord in equine, which showed that the freezing exerted few or no effect on the capacity of these cells in adhering and form colonies (Maia et al., 2017).

Generally, the present study showed that frozen cells in the different FS maintained the differentiation capacity for the adipogenic, osteogenic and chondrogenic lineages, as demonstrated in the qualitative evaluation and indicating that this function was well-preserved. However, the quantitative evaluation reveals that the osteogenic differentiation potential was inferior to the control group despite did not have differences among the FS (DMSO, DMSO+X-1000, and DMSO+Trehalose+X-1000). For the chondrogenic differentiation, only the DMSO+X-1000 showed a reduction in the differentiation potential to the control, but similar to the other treatments. Even with the exact mechanism of the pluripotency or multipotency has not been adequately explained (Naaldijk et al., 2012), it is known that the cell and tissue exposition to cryogenic temperatures alters many metabolic pathways. Overall, these modifications were characterized by the reduction in the activity of sodium and potassium pump, as well the alterations in the plasmatic membrane lipidic phase, and consequently interfering in the physiological function of enzymes and the precipitation of substances (Silva et al., 2017). We believe that these alterations could be the cause of the reduction in the differentiation potential of the eAT-MSCs after the different procedures of freezing investigated by our study.

Regarding the adipogenic differentiation, the frozen eAT-MSCs in all the treatments maintained the differentiation potential similar to the control. This finding showed a contrast to the observed in the osteogenic and chondrogenic differentiation, which did not affect the differentiation capacity of eAT-MSCs to adipocytes after slow freezing. This fact can be triggered by the existence of a higher predisposition of SC originated from the adipose tissue to effectively differentiated in adipocytes. The previous study (Fülber et al., 2016) reported that the ancestral microenvironment directs the “destination” cell upon differentiation when observed an elevated potential to chondrogenic differentiation when the SC used were originated from synovial tissue and not from the other sources. In parallel, the same cells had a low potential to adipogenic differentiation. Similarly, Koga et al. (2007) compared the differentiation capacity of SCs originated from the adipose tissue and the synovial membrane and observed a better chondrogenic differentiation capacity of SC from the synovial origin.

In conclusion, these findings confirmed the success of the slow freezing of eAT-MSCs in the presence of DMSO, demonstrated a high rate of cellular viability after the thawing. Moreover, the eAT-MSCs showed that the association of DMSO with the synthetic polymer X-1000 was extremely satisfactory concerning proliferation, colony formation capacity and cellular differentiation. Therefore, considering 1) the toxic effects caused to the cells by DMSO in the physiological and room temperature; 2) the loss of cells during the wash by the removal of DMSO before of the administration, and 3) the acceptable results of the association of DMSO with X-1000, we believe further investigations could be performed aiming to reduce the DMSO concentration, associated to the high concentrations of X-1000. Under this perspective, the possible reduction of the concentration of DMSO in the frozen protocols of SCs would eliminate the wash loss in the post-freezing cells, and consequently, minimizing the loss and the toxic effects observed during the previously cryopreserved SC transplantation.
